# Phylogenetic conservation of the interdependent homeostatic relationship of sleep regulation and redox metabolism

**DOI:** 10.1007/s00360-023-01530-4

**Published:** 2024-02-07

**Authors:** Aslihan Terzi, Keri J. Ngo, Philippe Mourrain

**Affiliations:** 1 Department of Psychiatry and Behavioral Sciences, Stanford University, Stanford, CA USA; 2 Department of Developmental Biology, Stanford University, Stanford, CA USA; 3 INSERM 1024, Ecole Normale Supérieure, Paris, France

**Keywords:** Sleep, Homeostasis, Redox, Metabolism, Zebrafish, Cavefish

## Abstract

Sleep is an essential and evolutionarily conserved process that affects many biological functions that are also strongly regulated by cellular metabolism. The interdependence between sleep homeostasis and redox metabolism, in particular, is such that sleep deprivation causes redox metabolic imbalances in the form of over-production of ROS. Likewise (and vice versa), accumulation of ROS leads to greater sleep pressure. Thus, it is theorized that one of the functions of sleep is to act as the brain’s “antioxidant” at night by clearing oxidation built up from daily stress of the active day phase. In this review, we will highlight evidence linking sleep homeostasis and regulation to redox metabolism by discussing (1) the bipartite role that sleep–wake neuropeptides and hormones have in redox metabolism through comparing cross-species cellular and molecular mechanisms, (2) the evolutionarily metabolic changes that accompanied the development of sleep loss in cavefish, and finally, (3) some of the challenges of uncovering the cellular mechanism underpinning how ROS accumulation builds sleep pressure and cellularly, how this pressure is cleared.

## Introduction

Sleep is an instrumental biological process in restorative functions, memory consolidation, cognitive abilities, and energy conservation (Appelbaum et al. 2010; Kayaba et al. 2017; Klinzing et al. 2019; Mullington et al. 2000). Using behavioural criteria, sleep has been described in all branches of the animal kingdom from invertebrate octopi, worms, and insects to vertebrates including fish and mammals (Campbell and Tobler 1984; Keene and Duboue 2018). These criteria include reduced mobility, increased arousal threshold, rapid reversibility, place preference/posture, and homeostatic regulation (Campbell and Tobler 1984; Jaggard et al. 2021). Despite the concentrated research on mammals, the current sleep definition lacks an all-inclusive explanation to cover its evolutionary-conserved role, lending support to the hypothesis that sleep serves a fundamental need, and the best way to define sleep would be to identify this need.

The neurological definition of sleep has been established since the 1950–60s to include the main electrophysiological hallmarks of human sleep recorded by polysomnography (PSG), combining electroencephalogram (EEG), electromyogram (EMG of voluntary muscles), electrocardiogram (ECG) and electrooculogram (EOG). Slow-wave sleep (SWS), the deepest stage of non-REM sleep (NREM), is characterized by slow, synchronized neocortical waves and low muscle activity. In contrast to NREM/SWS, Rapid Eye Movement sleep (REM), also known as Paradoxical Sleep (PS), has a core characteristic of muscle atonia with wake-like desynchronized EEG, cardiorespiratory irregularities, tremors, and episodic bursts of rapid eye movements. Until recently, these neurological sleep stages had only been reported in the more evolutionary-recent amniotic vertebrates: mammals and birds (Campbell and Tobler 1984; Leung and Mourrain 2018; Shein-Idelson et al. 2016). Our group has identified analogous cellular signatures of sleep in zebrafish including synchronous slow oscillation and paradoxical sleep activity, suggesting that neural and muscular patterns of NREM-REM sleep emerged at least 450 million years ago (Leung et al. 2019). To advance the understanding of conserved sleep need, the cellular and molecular functions of sleep need to be further studied. Having phylogenetically conserved behavior and neural sleep profile, fish provides a simpler vertebrate model for sleep studies that overcomes the challenges of studying sleep in mammalian systems. Some of these challenges include, but not limited to, animal handling and care, invasiveness of live imaging as well as behavioural tracking, and accessibility of deeper tissues and organs.

To this date, there is no united opinion on the function of sleep. From the earlier sleep research, the theories behind the function of sleep remain similar (Rechtschaffen 1998). Some prominent theories on the function of sleep include: (1) restorative theory, suggesting that sleep repairs the body through increased production of growth hormone, induced muscle repair and strengthened immune system (Rechtschaffen and Bergmann 2002), (2) memory consolidation theory, suggesting different stages of sleep contribute to various aspects of memory formation and organization (Diekelmann and Born 2010), (3) synaptic homeostasis hypothesis, suggesting that sleep is necessary to downscale and reset synaptic strength in the brain, preventing overload and maintaining optimal neuronal function (Tononi and Cirelli 2006), (4) energy conservation theory, suggesting that by reducing activity and metabolic rate during sleep, the body conserves energy that can be used during waking hours (Siegel 2005), and finally (5) brain plasticity and learning theory, suggesting that sleep promotes brain plasticity to enhance learning and adaptation to new experiences (Frank 2006). It must be noted that these theories are not mutually exclusive, and sleep likely serves multiple functions. Furthermore, these theories do not provide an explanation to the evolutionary-conserved roles of sleep.

Unquestionably, sleep is not solely for brain but also for the entire body. Chronic sleep deprivation systematically leads to weight gain and metabolic imbalances and sleep deprivation decreases antioxidant levels in rats and humans, leading to oxidative stress (D'Almeida et al. 1998; Everson et al. 2005; Ramanathan et al. 2002; Silva et al. 2004; Trivedi et al. 2017; Van Cauter et al. 2008). As sleep regulation and cellular sleep dynamics are highly conserved, so too may the interplay between sleep and redox homeostasis and the underlying mechanisms be similarly conserved across evolution. A theory for sleep, that also supports a restorative function for sleep, was proposed in which sleep functions essentially as an “antioxidant” for the brain where excess free radicals are removed during sleep (Reimund 1994). Wake periods consist of higher metabolic rates and higher brain activity and a selective metabolic shift occurs during sleep (DiNuzzo and Nedergaard 2017). During sleep, the body’s metabolic rate decreases and this reduction in metabolic activity could contribute to a lower oxidative environment along with an increased antioxidant capacity, decreasing overall free radicals and the oxidized substrates, that accumulated throughout the wake period due to high cellular metabolic activities. Although it is unlikely that the sole function of sleep is to clear oxidation from the brain, this theory points towards a direction for the new definition of sleep: what are the metabolic, particularly redox-related, implications of sleep?

Redox metabolism represents the reduction and oxidation (redox) reactions induced via oxidized molecular oxygen, or reactive oxygen species (ROS). ROS are highly reactive chemicals, enabling electron transfer from one molecule to another. The cellular redox state is determined by the balance between ROS production and the counteracting cellular antioxidant systems. Under physiological redox conditions, the production and elimination of ROS are in homeostasis and redox signalling is in action, during which proteins are post-translationally modified via oxidation to relay signal transduction (Dickinson and Chang 2011; Finkel 2011; Reczek and Chandel 2015). When the production and presence of ROS dominates the antioxidant capacity, cell undergoes oxidative stress, leading to damage to cellular components and causing aging, cancer, neurodegenerative diseases (Davalli et al. 2016; Park et al. 2008; Rama Rao et al. 2018; Weinberg et al. 2019). On the contrary, when ROS production is limited below physiological levels, disruption in redox signalling interferes with variety of cell functions including stem cell maintenance and differentiation, cell migration, axonal growth and guidance (Le Belle et al. 2011; Munnamalai et al. 2014; Somanna et al. 2016; Terzi et al. 2020). Hence, it is critical to maintain physiologically relevant levels of intracellular ROS for health and longevity.

The main cellular sources of ROS are (1) mitochondria, where electron-transport-chain (ETC) leads ROS production through leakage of electrons during oxidative phosphorylation, (2) NADPH oxidases (NOXes), which generate superoxide and hydrogen peroxide (H_2_O_2_) through transferring electrons from NADPH donors to oxygen, regulating both immune response and homeostatic ROS signalling (3) peroxisomes, which produce H_2_O_2_ as a byproduct during breakdown of metabolic reactions such as breakdown of fatty acids and (4) xanthine oxidase, which generates superoxide and H_2_O_2_ as a byproduct of oxidative hydroxylation of hypoxanthine in purine metabolism; (5) cytochrome P450 enzymes, a group of heme monooxygenases, which catalyse the metabolism of endogenous and exogenous molecules by electron transfer through NADPH cofactor, and finally (6) endoplasmic reticulum, which generates ROS in response to misfolded proteins (De Almeida et al. 2022).

**Fig. 1:**
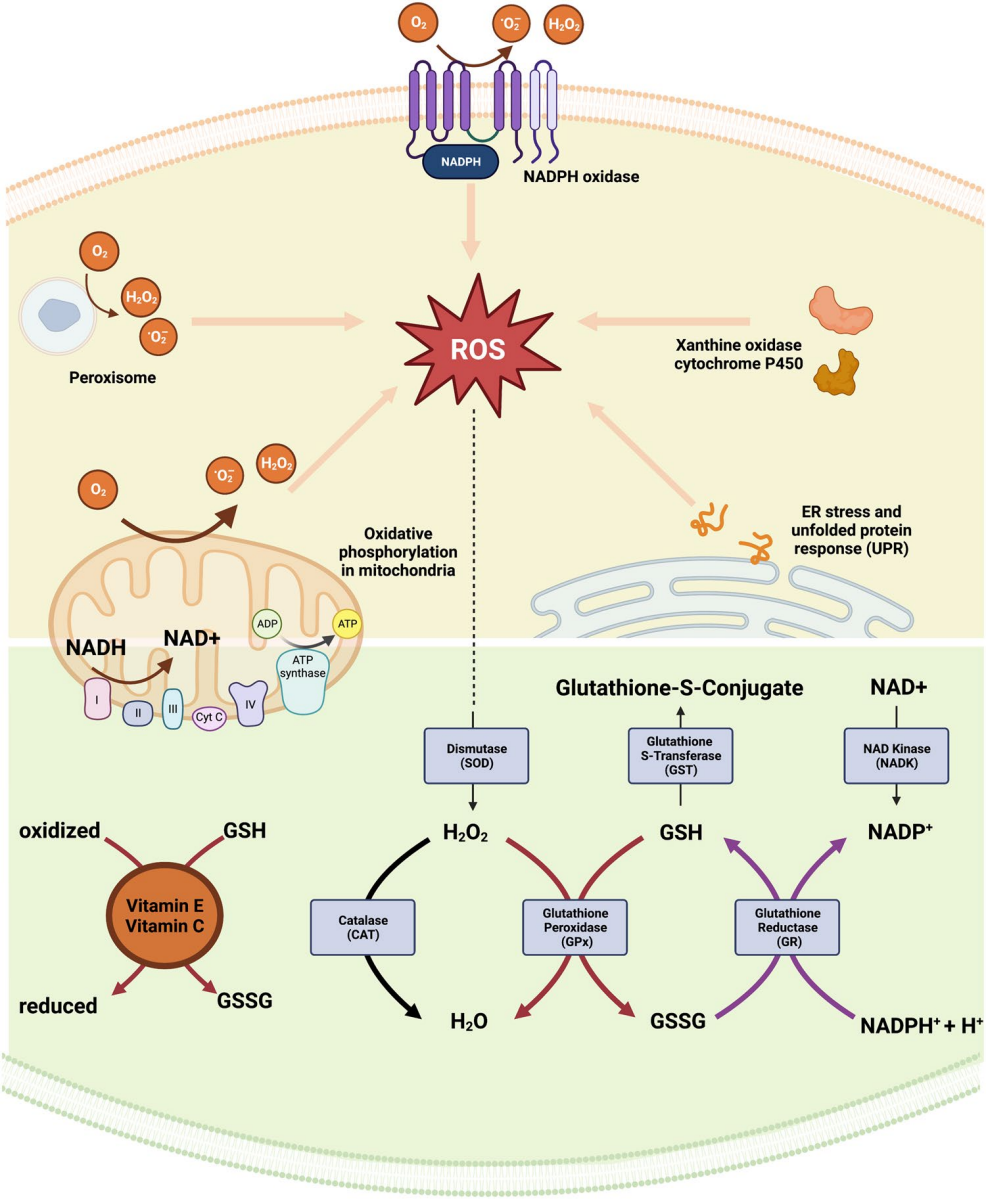
Summary of major intracellular ROS sources and antioxidant systems. The major intracellular ROS sources (upper panel) include: NADPH oxidases, peroxisomes, oxidative phosphorylation in mitochondria, xanthine oxidases, and the endoplasmic reticulum stress response towards unfolded proteins (UPR). The major antioxidant systems (lower panel) include enzymatic (grey): catalase, superoxide dismutase (SOD), glutathione peroxidases; and nonenzymatic (orange): glutathione (GSH), vitamin C, and vitamin E

The counteracting cellular antioxidant systems include both enzymatic, such as catalase, superoxide dismutase (SOD), glutathione peroxidase, and nonenzymatic, such as glutathione (GSH), vitamin C, vitamin E, members (Haida and Hakiman 2019). In addition, uncoupling proteins (UCPs), located in the inner mitochondrial membrane, prevents ROS accumulation, and lowers the mitochondrial membrane potential, and their overexpression was found to counteract oxidative stress (Barreiro et al. 2009; Hirschenson et al. 2022). Finally, autophagy and/or mitophagy mechanisms act against cellular oxidative stress (Filomeni et al. 2015). Increased ROS production triggers autophagy to mediate clearing of ROS and oxidative damage (Chen et al. 2007). As mitochondria are major source of ROS, chronic impairment of any kind of mitochondrial function overproduces ROS and triggers a self-removal signal, or mitophagy, to eliminate the further oxidative environment (Schofield and Schafer 2021). Redox metabolism is not only maintained by ROS producing enzymes and antioxidants but also by nicotinamide adenine dinucleotide (NAD +) and its metabolites. NAD + and NAD + -related metabolites, NADH, NADP + and NADPH, are crucial in energy metabolism, DNA repair, epigenetic modifications, inflammation and circadian rhythms (Xie et al. 2020). NAD + and its metabolites serve as co-enzymes for redox reactions, relaying oxidative and reductive signalling between molecules. Coupled NAD + /NADH redox exert their main effect in mitochondria, by serving as an electron donor through ETC for oxidative phosphorylation and producing cellular ROS (Li and Sauve 2015), but they also exhibit protective effects by enhancing GSH levels and the activity of antioxidant enzymes (Wang et al. 2014). NADPH, also, serves both as an electron donor for NADPH oxidases, contributing to cellular ROS production, and as a reductive power for antioxidant defence by transferring electrons from enzymatic antioxidants (Bedard and Krause 2007; Bradshaw 2019). The major intracellular redox players are summarized in Fig. 1.

At night, oxidized molecules are either repaired or replaced in plants (Bechtold et al. 2004). Similarly, in animals, wake and sleep loss elevate mitochondrial ROS in dorsal fan-shaped body (dFB) neurons (Kempf et al. 2019). Like the night phase in plants, sleep has been involved in toxin elimination, DNA repair, and infection defense (Mourrain and Wang 2019). Further, recent studies in flies showed that (1) short-sleeping mutants are extremely sensitive to oxidative stress, suggesting that a key function of sleep is to defend against oxidative stress (Hill et al. 2018); (2) ROS accumulation builds sleep pressure sensed in specific neurons that promote sleep, which in turn dissipate ROS burden and return the mitochondrial NAD + /NADH ratio to baseline (Kempf et al. 2019). Although the exact mechanisms have not been discovered yet, accumulation of ROS during the active phase or day, builds up sleep pressure that is cleared during inactive phase, or night, thus, getting the body and brain ready for the prospective day.

In the forthcoming sections, we will focus on the current knowledge on the relationship between redox metabolism and sleep homeostasis mainly in zebrafish and cavefish, where such processes are well conserved, as vertebrate models for human studies, and discuss the implications of this relationship on the universal definition of sleep.

## Behavioural and neural definition of sleep is conserved in fish

Zebrafish are diurnal animals and exhibit a recurring pattern of sleep and wakefulness, similar to humans. Zebrafish experience consolidated periods of sleep at night. This sleep state is characterized behaviourally by reduced locomotor activity, decreased responsiveness to stimuli, and homeostatic regulation (Yokogawa et al. 2007; Zhdanova et al. 2001; Zhdanova. 2006). Zebrafish also have sleep brain dynamics analogous to mammals, including Slow Bursting Sleep (SBS) and Propagating Wave Sleep (PWS). SBS shares many commonalities with NREM-Slow Wave Sleep by exhibiting synchronous, slow and high amplitude oscillations of the telencephalic neurons occurring in an overall background of reduced brain activity, low muscle tone, and reduced but steady cardiovascular activity (Leung et al. 2019). PWS is a sleep state sharing many features with REM/Paradoxical Sleep, including pontine activation, ponto-midbrain-telencephalic wave propagation, rostro-caudal voluntary muscle atonia propagation, heart beat and breathing arrhythmia as well as wake-like activity of the telencephalon (Leung et al. 2019). The regulation of sleep involves at least two key processes in zebrafish as it does in other organisms: (1) the circadian process, which aligns sleep patterns with the natural 24-h day and night cycle, and (2) the homeostatic process, which increases the urge to sleep based on the duration of prior wakefulness. Here, we will engage in discussion on homeostatic regulation of sleep only in relation to redox metabolism.

## Sleep-modulating neuropeptides are conserved in zebrafish

Neuropeptides that control sleep–wake states in mammals are conserved in fish as well. Galanin, an important neuropeptide in energy homeostasis and sleep regulation, is also required for homeostatic sleep rebound following sleep deprivation in zebrafish larvae (Martinelli et al. 2021; Reichert et al. 2019). Neuropeptide Y (NPY), another highly conserved neuropeptide, contributes to controlling energy homeostasis, anxiety and sleep; exhibiting dual impact on sleep–wake behaviours in mammals (Hsieh et al. 2013; Shen et al. 2022). In zebrafish, NPY promotes sleep via inhibition of noradrenergic signaling and loss-of-function mutations of NPY resulted in decreased sleep (Singh et al. 2017). Neuromedin U, a key neuropeptide regulating feeding, energy metabolism and insulin secretion, has been shown to inhibit sleep in zebrafish, similar to its function in rats (Chiu et al. 2016; Wren et al. 2002). The hypocretin/orexin neuropeptides are essential for the maintenance of wakefulness and the suppression of REM sleep, and zebrafish possess a functional hcrt-pineal gland circuit, connecting the hcrt and melatonin systems together in sleep consolidation (Appelbaum et al. 2009). In addition to neuropeptides, melatonin, a naturally occurring hormone, is produced in the pineal gland at night and is also required for circadian regulation of zebrafish sleep (Kazimi and Cahill 1999; Zhdanova et al. 2001). Melatonin’s ability to facilitate sleep is evolutionarily conserved as it is widely used as an over-the-counter sleep aid. Finally, adenosine regulates homeostatic sleep by creating sleep pressure and inducing sleep as a result of accumulation during prolonged wakefulness (Wigren et al. 2007). The role of adenosine in sleep homeostasis remains a topic of controversy in different species. In Drosophila, caffeine-mediated reduction and fragmentation of sleep did not depend on adenosine receptor (Wu et al. 2009). Similarly, adenosine receptor knock-out mice did not exhibit any defects in sleep homeostasis (Stenberg et al. 2003). However, more recent evidence in mice suggests that intracellular adenosine mediates sleep homeostasis through glial-neural circuits; while homeostatic sleep drive was enhanced in glial-deficient adenosine, neural-deficient adenosine did not influence sleep drive (Bjorness et al. 2016). In zebrafish, adenosine was reported to trigger sleep and prevent activity during the day but not at night (Mourrain lab unpublished data; Gandhi et al. 2015).

## Sleep-modulating neuropeptides, hormones and their connection to redox metabolism

Sleep–wake regulating neuropeptides are implicated in cellular redox metabolism. For instance, elevated NPY enhances cellular redox potential by increasing NADPH and the production of NPY itself is mediated by ROS (Raghuraman et al. 2011; Schwetz et al. 2013). Another example is that the loss of functional galanin causes mitochondrial oxidative stress in mice (Boal et al. 2022). In addition, increased orexin A/hypocretin 1 peptide causes mitochondrial impairment and dysfunction, contributing to increased cellular ROS (Li et al 2020). Lastly, adenosine alleviates oxidative stress by increasing expression levels of an essential antioxidant gene, nuclear factor (erythroid‑derived 2)‑like 2 (Nrf2) (Gholinejad et al. 2018), suggesting that adenosine and oxidative burden could act together to drive sleep and bring redox balance back to homeostatic levels.

A direct relationship between ROS and sleep-regulating neuropeptides remain to be elucidated to better understand the mechanism of action of ROS on these neuropeptides, however, there is evidence from *C. elegans* studies that mitochondrial-produced ROS can modulate neuropeptide release: in cholinergic motor neurons, neuropeptide-like protein (NLP-21) secretion was inhibited in two-different mutant types, both mutants causing enhanced mitochondrial ROS production via different mechanisms (Zhao et al. 2018). On the other hand, mitochondria-derived ROS increased secretion of neuropeptide FMRP-like peptide (FLP-1) in AIY interneurons, which in turn induced a major antioxidant mechanism, Nrf2, to eliminate excess ROS and ameliorate oxidative stress (Jia and Sieburth 2021). Hence, depending on cell type and upstream activators, ROS can have different effects on neuropeptide secretion. Overall, major cell-to-cell signalling molecules regulating sleep–wake behavior in mammals and zebrafish have also been implicated in oxidative stress/redox homeostasis, which may involve positive as well as negative feedback loops for further regulation of redox control and neuropeptide production and/ or release.

Melatonin is the first hint connecting redox metabolism to sleep function through its functions beyond regulating circadian rhythms. Melatonin is quite a peculiar compound as the function of melatonin has evolved and diversified over 3 billion years. Melatonin is believed to serve as an antioxidative agent, evolving in photosynthetic bacteria and acquiring new roles in circadian regulation and sleep in the present day (Manchester et al. 2015; Tan et al. 2013). While pineal melatonin regulates the circadian rhythms, extra pineal melatonin performs various functions beyond its primary role, which includes acting as an antioxidant, stimulating the production of endogenous antioxidant enzymes, scavenging free radicals, and playing a homeostatic role within mitochondria (Aranda‐Martínez et al. 2022; Gandhi et al. 2015; Zhdanova et al. 2001). It has been shown that melatonin is capable of indirectly scavenging free radicals by regulating the activity and expression of other antioxidant systems in both plant and mammals (Bidabadi et al. 2020; Morvaridzadeh et al. 2020; Nogués et al. 2006). In zebrafish, like in other animals, melatonin also maintains the redox balance by regulating the ratio of reduced glutathione to oxidized glutathione (GSH/GSSG), reducing lipid peroxidation, and enhancing the activity and expression of other antioxidant enzymes such as SOD and catalase (Duarte et al. 2023; Lunkes et al. 2021; Yan et al. 2022). Furthermore, melatonin administration alleviated the reduction in catalase, glutathione peroxidase and SOD activity following sleep-deprivation and enhanced GSH/GSSG ratio levels to normal (Alzoubi et al. 2016). In zebrafish, there is only one study that utilized melatonin treatment in sleep-deprived animals, and showed that melatonin treatment did not rescue the learning performance in zebrafish (Pinheiro-da-Silva et al. 2018). However, this study lacks in-depth biochemical measures at the cellular level to assess any improvement in the cellular effects of sleep deprivation. Finally, in a zebrafish model of Parkinson’s disease (PD), a neurodegenerative disorder where sleep disturbances are among the most prevalent symptoms, melatonin administration restored the sleep–wake cycles of animals with impaired rhythm and dysfunctional mitochondria (Aranda-Martínez et al. 2023; Mattis and Sehgal 2016). This is similar to studies where melatonin administration prevents sleep loss in PD patients and confers protective effects on mitochondria in mice and rat models of PD (Daneshvar Kakhaki et al. 2020; López et al. 2017; Paul et al. 2018). Thus, melatonin has a bipartite role in sleep through circadian and homeostatic redox regulation.

## Role of redox metabolism in sleep homeostasis

**Fig. 2:**
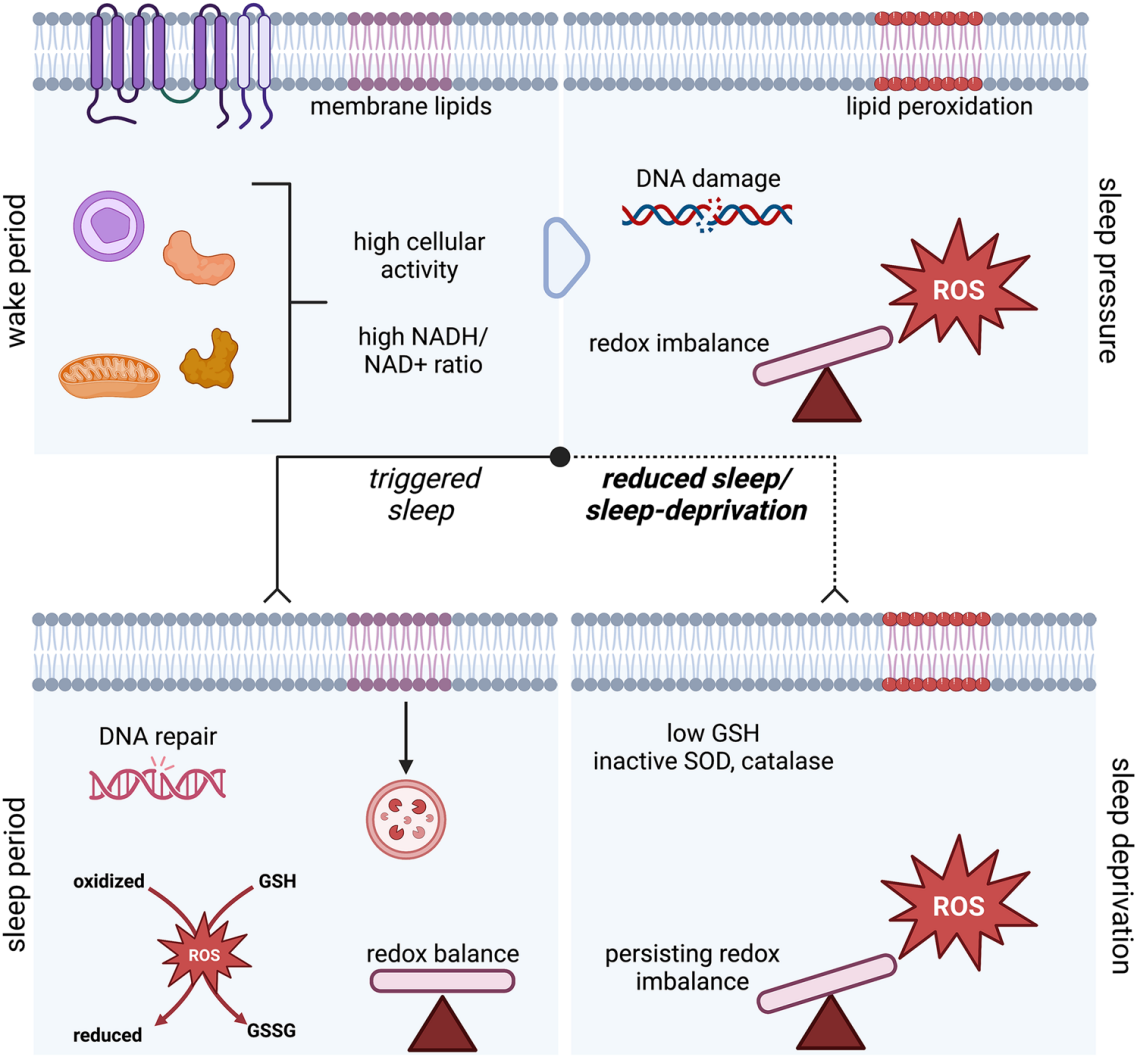
Schematics of redox-related changes during sleep–wake cycle. ROS accumulates from high cellular activity and increased NADH during the wake period (top left), creating sleep pressure (top right) in the form of membrane lipid peroxidation, redox imbalances and DNA damage. The effects of the wake period are ameliorated during the sleep period (bottom left), where DNA damaged during the day period is repaired and membrane lipids and redox systems are replenished and returned to homeostatic levels. Inadequate sleep and sleep deprivation (bottom right) worsen the redox imbalance with lower antioxidant systems (GSH, SOD, catalase) that cannot prevent further ROS accumulation

It is well-documented that during prolonged sleep deprivation, ROS production is elevated in multiple species and in multiple organs including different regions of the brain (hippocampus, frontal cortex, cerebellum, neocortex), liver, heart, gut, and skeletal muscle (Kempf et al. 2019; Rodrigues et al. 2018; Vaccaro et al. 2020; Villafuerte et al. 2015). However, the source of this over-produced ROS following sleep-deprivation is currently unknown. The possible sources are: (1) decreased expression and activity of cellular antioxidant systems (discussed above), or (2) increased production of ROS through major sources, such as mitochondria, that overcome the cellular antioxidant capacity, or (3) both. Mitochondria undergo several ultrastructural and biochemical changes after sleep deprivation. Mitochondrial size and density increases, fusion/fission dynamics are dysregulated, ETC efficiency decreases, and ROS production increases (De Vivo et al. 2016; Flores et al. 2022; Lu et al. 2021). Mitochondria-derived ROS turns on the dFB neurons, which are more excitable with higher sleep pressure in fruit flies. dFB neurons are activated through the voltage-gated potassium channel Shaker and its subunit Hyperkinetic, and loss-of-function mutations in either protein cause insomnia (Bushey et al. 2007; Cirelli et al. 2005; Kempf et al. 2019). Hyperkinetic has a redox-sensing capability via its binding to NADPH in its active site; as sleep pressure increases, mitochondria ETC enhances ROS production, which in turn oxidizes NADPH to NADP + . As a result, Hyperkinetic is more likely converted to its NADP + -bound form, such that A-type potassium current flows through Shaker with slower inactivation, and thus enhancing the activity of sleep-control neurons (Kempf et al. 2019). Similarly, loss of voltage-gated potassium channel *kcna2* and Na + /K + pump *atp1a3* genes in zebrafish reduced sleep, and it is highly likely that redox metabolism and sleep are mechanically connected in zebrafish as in fruit flies (Barlow et al. 2023; Srdanovic et al. 2017). In zebrafish, intracellular NAD(H) levels were shown to be reduced in the absence of Letm1, a conserved mitochondrial cation exchanger which follows diurnal rhythms together with NAMPT, the key enzyme in NAD + production (Dao et al. 2022). Finally, Parp1, which is both an NAD + consuming and a DNA repair enzyme, can trigger sleep in zebrafish and adult mice. Zada et al., showed that neuronal DNA damage, which builds up during the day, increases sleep pressure and that Parp1 activity promotes sleep to facilitate efficient DNA repair and clearing of the pressure (Zada et al. 2021). Thus, the redox metabolites NAD + /NADH and NADP + /NADPH serve as a link between cellular metabolism and sleep. It has been acknowledged that during the active phase, high cellular activity concentrates ROS from different sources, leading to increased sleep drive. During sleep, antioxidant mechanisms take over to counteract the ROS accumulation and restore the cellular redox balance. When the ROS burden cannot be alleviated because of reduced or lack of sleep, the redox imbalance persists and causes cellular damage. The metabolic regulation of sleep and the redox state is summarized in Fig. 2.

## *Astyanax mexicanus*: lessons to be learned from fish that sleep less

*Astyanax mexicanus*, commonly known as the Mexican tetra, exhibits two-different morphotypes: surface- and cave-dwelling populations (Jeffery 2020). These populations have diverged due to their contrasting habitats and environmental conditions. The surface populations reside in rivers and streams with access to nutrients, while the cave populations inhabit underground cave systems, having adapted to scarce nutrient environments (Rohner 2018). The surface populations have retained their pigmentation and functional eyes, allowing them to thrive in lighted environments. In contrast, the cave populations have undergone evolutionary changes, resulting in reduced pigmentation, and degenerated or non-functional eyes, as they inhabit dark cave environments with limited or no access to light (Krishnan and Rohner 2017; Moran et al. 2015). These adaptations reflect the selective pressures acting on each population, such as the surface populations adapting to visual cues and the cave populations relying on other sensory mechanisms for survival in the darkness. The study of these distinct populations provides valuable insights into evolutionary processes, as well as metabolic and molecular adaptations to extreme environments.

Interestingly, cavefish exhibited 80% reduction in their sleep compared to surface-dwelling populations, thus becoming a robust model for understanding evolutionary changes in sleep (Jaggard et al. 2018; Keene and Appelbaum 2019; Yoshizawa et al. 2015). Two interconnected mechanisms have been reported to affect the sleep reduction in cavefish populations: sensory input and the wake-promoting Hcrt neuropeptide. The lateral line of fish consists of mechanosensory organs called neuromasts, and the number, size and sensitivity of neuromasts to sensory stimuli are more profound in cavefish than in surface-dwelling populations (Yoshizawa et al. 2014). Targeted ablation of neuromasts in cavefish enhanced their sleep, suggesting that increased sensory input is responsible for sleep loss (Jaggard et al. 2017). In addition, the Hcrt/orexin network is conserved in Mexican tetra, and loss of Hcrt function in cavefish restores sleep to levels similar to those in surface fish (Jaggard et al. 2018). Furthermore, ablating mechanosensory lateral line reduced Hcrt levels in cavefish, implying that the recognition of sensory cues through the lateral line plays a crucial role in promoting Hcrt signalling (Jaggard et al. 2018), thereby maintaining wakefulness. Despite the given role of sensory responsiveness in sleep reduction, it is noteworthy that lateral line ablation did not have an impact on sleep in four separate populations of cavefish (Jaggard et al. 2017). This finding implies that unique mechanisms likely govern the evolutionary process of sleep loss in independently derived cavefish populations, or there are other commonly evolved mechanisms that are important in sleep regulation, that have yet to be studied.

Cavefish face extreme conditions in their natural habitats such as long periods of nutrient deprivation, which in turn triggers chronic stress. As opposed to other organisms, for which these conditions would be deleterious, cavefish overcome the environmental challenges and maintain physiological health via metabolic adaptations, potentially contributing to reduced sleep phenotype in cavefish. Compared to surface-dwelling populations, cavefish acquired insulin resistance and elevated blood glucose in multiple cave populations to develop metabolic resilience (Riddle et al. 2018). Decreasing sleep duration significantly reduced insulin sensitivity in healthy individuals, and sleep loss is a risk factor for insulin resistance and type 2 diabetes in humans, suggesting insulin-mediated metabolic changes could have impact on sleep regulation (Buxton et al. 2010; Spiegel et al. 2005). Furthermore, redox alterations have bidirectional role in developing insulin resistance: H_2_O_2_ has been shown in mice to attenuate insulin resistance while chronic ROS production via mitochondria and NOXes contribute to development of insulin resistance by pro-inflammatory cytokines (Loh et al. 2009; Tiganis 2011). Unlike the surface morphotype, cavefish have lower levels of ROS and enhanced antioxidant activities, which could contribute to reduced need for sleep and weaker oxidative response to stress conditions. Key antioxidant genes involving GSH metabolism were upregulated and the major cellular antioxidant, GSH, as well as vitamin C, were increased in the liver and brain of cave populations, but not in surface-dwelling populations (Krishnan et al. 2020; Medley et al. 2022). Moreover, under stress conditions of prolonged starvation, cavefish exhibited lower cytoplasmic ROS compared to surface fish (Medley et al. 2022), indicating that ROS accumulation is likely to be at a lesser extent in cavefish populations, supporting the hypothesis that ROS accumulation creates sleep pressure and ROS is cleared during sleep.

## Concluding remarks

Despite being evolutionarily conserved across all animals, the core physiological function of sleep remains unclear. The imbalance between the antioxidant defense system and the generation of oxidants creates oxidative stress which can further cellular damage and compound adverse issues from chronically impaired sleep such as obesity and even neurodegenerative disorders. Redox metabolism, like sleep, is well conserved such that from plants to animals, it has been found that oxidized molecules are dissipated at night to facilitate processes like DNA repair. Here, we primarily focus on zebrafish, a highly amenable model in which sleep, and metabolic mechanisms are highly conserved, to delineate the reciprocal relationship between sleep homeostasis and redox metabolism.

As mentioned above, it is well documented across species that prolonged sleep deprivation increases susceptibility to oxidative stress in the form of increased mitochondrial ROS which in turn increases sleep pressure. During sleep, this pressure is ameliorated by removing scavenging free radicals with antioxidant enzymes which can be regulated with sleep–wake modulators like melatonin. Concordantly, some species, like the cavefish highlighted in this review, have evolved metabolic evolutionary changes different from their surface-dwelling counterparts to increase their resilience to oxidative stress via lower ROS and higher antioxidant levels. If a key function of sleep is to defend against oxidative stress accumulated during the wake period, these metabolic changes to prevent oxidative stress in cavefish may be contributing to their shorter sleep phenotype and might mean that differences in sleep between animals or species is due in part to metabolic differences. In addition, it is possible that evolution led to shared redox-sensing channels (e.g., Hyperkinetic and Shaker) to mechanically link sleep-active neurons and redox metabolism in order to more efficiently serve a common fundamental need. This fundamental need requires identification to truly define sleep in an explanation that accounts for the many evolutionarily conserved roles of sleep, such as in redox metabolism as we covered here.

In this review, we cover the significance and the mechanisms of redox metabolism and sleep homeostasis and how they are connected. However, there are challenges remaining to pinpoint the cellular mechanisms that explain where and how exactly ROS accumulation builds sleep pressure and how precisely this sleep pressure is cleared. We focus particularly on to explore how this ROS accumulation contributes to sleep pressure buildup and the exact mechanisms by which this sleep pressure is cleared as these questions remain unknown in this group of organisms. Metabolic homeostasis and the cellular redox systems are delicate systems that differ in subcellular compartments and depend on the physiological condition of the animal/cell (e.g., circadian rhythm, cell cycle stage, disease pathology). Due to these finer differences, non-targeted treatments of reactive oxidant radicals which are highly specific in their signalling, may fail as a remedy if subcellular precision at specific timepoints is necessary. Therefore, the study of intracellular mechanisms at the interplay of redox metabolism and sleep–wake regulation remains a difficult endeavor. Future studies may require precise targeting of both systems in tandem to fully elucidate this key function of sleep that maintains metabolic homeostasis.
